# Supplementary material for: Variability in Biological Activities of Satureja montana Subsp. montana and Subsp. variegata Based on Different Extraction Methods
Source: Antibiotics (Basel). 2022 Sep 11;11(9):1235. doi: 10.3390/antibiotics11091235 (PMC9495055; doi:10.3390/antibiotics11091235)
Supplement: Supplementary file 1 [file antibiotics-11-01235-s001.zip › Supplementary Table S1.pdf]

Supplementary Table S1. Number of microorganisms (log CFU/mL) during contact with MIC value of antimicrobials.

Essential oil										
Microorganism	*S. montana* subsp.	Contact time (hours)								
		0	1	2	3	4	6	12	18	24
B. cereus	subsp. *montana*	6.1	5.1	4.4	3.0	1.8	1.0	0.0	0.0	0.0
	subsp. *variegata*	6.1	5.4	3.3	2.5	1.3	0.0	0.0	0.0	0.0
S. aureus	subsp. *montana*	5.8	3.1	2.3	1.9	1.5	1.05	0.0	0.0	0.0
	subsp. *variegata*	5.8	5.5	2.7	2.3	1.9	0	0.0	0.0	0.0
E. faecalis	subsp. *montana*	6	4.9	4.2	3.8	2.6	1.2	0.0	0.0	0.0
	subsp. *variegata*	6	5.5	5.1	4.5	2.3	0.0	0.0	0.0	0.0
E. coli	subsp. *montana*	6	4.8	4.0	3.4	2.1	1.4	0.0	0.0	0.0
	subsp. *variegata*	6	4.7	3.7	2.6	1.2	0.0	0.0	0.0	0.0
S. Typhimurium	subsp. *montana*	6.3	5.7	4.8	2.9	2.2	1.7	0.0	0.0	0.0
	subsp. *variegata*	6.3	3.9	2.2	1.1	0.0	0.0	0.0	0.0	0.0
		0	3	6	9	12	18	24	48	72
S. cerevisiae	subsp. *montana*	5.7	5.3	4.1	3.1	2.1	1.3	0.0	0.0	0.0
	subsp. *variegata*	5.7	3.6	3.2	2.1	2.0	0.0	0.0	0.0	0.0
C. albicans	subsp. *montana*	5.9	5.5	4.0	3.4	2.7	1.05	0.0	0.0	0.0
	subsp. *variegata*	5.9	4.2	4.1	3.7	3.4	0.0	0.0	0.0	0.0
Hydrolate										
		Contact time (hours)								
		0	3	6	9	12	18	24	48	72
S. cerevisiae	subsp. *montana*	5.7	3.8	3.7	3.5	2.7	2.2	1.2	0.0	0.0
	subsp. *variegata*	5.7	4.3	4.0	3.7	3.5	2.4	1.4	0.0	0.0
C. albicans	subsp. *montana*	5.9	5.1	4.9	4.8	3.1	3.0	2.7	0.0	0.0
	subsp. *variegata*	5.9	4.7	4.4	4.3	3.4	2.9	2.2	0.0	0.0
SWE										
		Contact time (hours)								
		0	1	2	3	4	6	12	18	24
B. cereus	subsp. *montana*	6.1	5.5	3.9	3.5	3.3	1.9	0.0	0.0	0.0
	subsp. *variegata*	6.1	5.8	5.5	5.5	5.3	2.7	1.1	0.7	0.0
UAE-MeOH										
		Contact time (hours)								
		0	3	6	9	12	18	24	48	72
S. cerevisiae	subsp. *variegata*	5.7	4.1	3.1	2.0	1.05	0.0	0.0	0.0	0.0
C. albicans		5.9	5.3	5.0	4.7	4.4	2.4	1.1	0.0	0.0
