# Supplementary material for: Variability in Biological Activities of Satureja montana Subsp. montana and Subsp. variegata Based on Different Extraction Methods
Source: Antibiotics (Basel). 2022 Sep 11;11(9):1235. doi: 10.3390/antibiotics11091235 (PMC9495055; doi:10.3390/antibiotics11091235)
Supplement: Supplementary file 1 [file antibiotics-11-01235-s001.zip › Supplementary Table S2.pdf]

Supplementary Table S2. The "goodness of fit" kinetics models.

Microorganism	*S. montana* subsp.	χ^2	RMSE	MBE	MPE	SSE	AARD	r^2	Skew	Kurt	Mean	StDev	Var
Essential oil													
B. cereus	*montana*	0.04	0.18	-0.03	3.68	0.29	1.38	0.99	-0.42	-0.27	-0.03	0.19	0.04
	variegata	0.07	0.24	-0.04	3.52	0.52	1.48	0.99	-0.1	1.74	-0.04	0.25	0.07
S. aureus	*montana*	0.07	0.24	-0.06	5.72	0.49	1.9	0.98	-0.31	-1.54	-0.06	0.25	0.06
	variegata	0.18	0.4	-0.03	8.7	1.42	2.89	0.97	-0.01	-0.58	-0.03	0.42	0.18
E. faecalis	*montana*	0.09	0.29	-0.05	5.16	0.71	2.24	0.98	0.63	-0.69	-0.05	0.3	0.09
	variegata	0.05	0.21	-0.02	2.15	0.4	1.22	0.99	0.51	1.93	-0.02	0.22	0.05
E. coli	*montana*	0.08	0.26	-0.06	4.52	0.56	2.06	0.99	0.38	-0.81	-0.06	0.27	0.07
	variegata	0.41	0.6	0.34	17.61	2.23	4.09	0.95	0.36	-0.99	0.34	0.53	0.28
S. Typhimurium	*montana*	0.07	0.25	-0.03	5.76	0.57	1.83	0.99	0.94	0.56	-0.03	0.27	0.07
	variegata	0.07	0.24	-0.05	4.14	0.51	1.62	0.99	-0.43	0.96	-0.05	0.25	0.06
S. cerevisiae	*montana*	0.06	0.22	-0.04	3.92	0.43	1.38	0.99	-0.99	2.65	-0.04	0.23	0.05
	variegata	0.24	0.47	-0.08	8.29	1.89	3.62	0.95	-0.09	-1.22	-0.08	0.49	0.24
C. albicans	*montana*	0.11	0.31	-0.05	4.53	0.83	2.08	0.98	-0.4	0.14	-0.05	0.32	0.1
	variegata	0.35	0.55	-0.03	7.33	2.75	3.3	0.94	1.79	4.4	-0.03	0.59	0.34
Hydrolate													
S. cerevisiae	*montana*	0.21	0.43	-0.07	8.7	1.64	3.36	0.95	-0.35	-1.11	-0.07	0.45	0.21
	variegata	0.17	0.39	-0.06	6.93	1.34	3.05	0.96	0.6	-0.55	-0.06	0.41	0.17
C. albicans	*montana*	0.25	0.47	-0.06	8.71	1.99	3.77	0.95	-0.03	-1.16	-0.06	0.5	0.25
	variegata	0.18	0.4	-0.06	6.95	1.41	3.29	0.96	0.36	-1.18	-0.06	0.42	0.18
SWE													
B. cereus	*montana*	0.15	0.36	-0.08	5.23	1.15	2.66	0.98	0.06	-0.43	-0.08	0.38	0.14
	variegata	0.1	0.29	0.00	9.27	0.77	2.21	0.99	-0.33	-0.37	0.00	0.31	0.1
UAE-MeOH													
S. cerevisiae	*variegata*	0.09	0.29	-0.07	4.36	0.69	2.1	0.98	-0.32	-1.28	-0.07	0.29	0.09
C. albicans		0.06	0.22	-0.02	3.69	0.44	1.56	0.99	1.19	1.53	-0.02	0.23	0.05

χ^2, reduced chi-square; RMSE, root mean square error; MBE, mean bias error; r^2, coefficient of determination; Skew, skewness; Kurt, kurtosis; Mean, mean of the residuals; StDev, standard deviation of the residuals; Var, variance of the residuals;
